# Uncoupling protein-2 mRNA expression in mice subjected to intermittent hypoxia*

**DOI:** 10.1590/S1806-37132015000004414

**Published:** 2015

**Authors:** Luciana Rodrigues Vieira, Denis Martinez, Luiz Felipe Forgiarini, Darlan Pase da Rosa, Gustavo Alfredo Ochs de Muñoz, Micheli Fagundes, Emerson Ferreira Martins, Carolina Caruccio Montanari, Cintia Zappe Fiori

**Affiliations:** Federal University of Rio Grande do Sul, Porto Alegre, Brazil. Graduate Program in Medicine: Medical Sciences, Federal University of Rio Grande do Sul, Porto Alegre, Brazil; Federal University of Rio Grande do Sul, School of Medicine, Porto Alegre, Brazil. Federal University of Rio Grande do Sul School of Medicine, Porto Alegre, Brazil; Faculdade Anhanguera de Pelotas, Pelotas, Brazil. Faculdade Anhanguera de Pelotas, Pelotas, Brazil; Lutheran University of Brazil, Canoas, Brazil. Faculdade Cenecista de Bento Gonçalves, Bento Gonçalves; and Collaborating Professor, Lutheran University of Brazil, Canoas, Brazil; Federal University of Rio Grande do Sul, Porto Alegre, Brazil. Graduate Program in Child and Adolescent Health, Federal University of Rio Grande do Sul, Porto Alegre, Brazil; Federal University of Rio Grande do Sul, Porto Alegre, Brazil. Graduate Program in Health Sciences: Cardiology and Cardiovascular Sciences, Federal University of Rio Grande do Sul, Porto Alegre, Brazil; Federal University of Rio Grande do Sul, Porto Alegre, Brazil. Graduate Program in Health Sciences: Cardiology and Cardiovascular Sciences, Federal University of Rio Grande do Sul, Porto Alegre, Brazil; Federal University of Rio Grande do Sul, Porto Alegre, Brazil. Graduate Program in Medicine: Medical Sciences, Federal University of Rio Grande do Sul, Porto Alegre, Brazil; Federal University of Rio Grande do Sul, Porto Alegre, Brazil. Graduate Program in Health Sciences: Cardiology and Cardiovascular Sciences, Federal University of Rio Grande do Sul, Porto Alegre, Brazil

**Keywords:** Blood glucose, Sleep apnea syndromes, Pancreas, Glucagon-secreting cells

## Abstract

**Objective:**

To investigate the effect of intermittent hypoxia-a model of obstructive sleep apnea (OSA)-on pancreatic expression of uncoupling protein-2 (UCP2), as well as on glycemic and lipid profiles, in C57BL mice.

**Methods:**

For 8 h/day over a 35-day period, male C57BL mice were exposed to intermittent hypoxia (hypoxia group) or to a sham procedure (normoxia group). The intermittent hypoxia condition involved exposing mice to an atmosphere of 92% N and 8% CO_2_ for 30 s, progressively reducing the fraction of inspired oxygen to 8 ± 1%, after which they were exposed to room air for 30 s and the cycle was repeated (480 cycles over the 8-h experimental period). Pancreases were dissected to isolate the islets. Real-time PCR was performed with TaqMan assays.

**Results:**

Expression of UCP2 mRNA in pancreatic islets was 20% higher in the normoxia group than in the hypoxia group (p = 0.11). Fasting serum insulin was higher in the hypoxia group than in the normoxia group (p = 0.01). The homeostasis model assessment of insulin resistance indicated that, in comparison with the control mice, the mice exposed to intermittent hypoxia showed 15% lower insulin resistance (p = 0.09) and 21% higher pancreatic β-cell function (p = 0.01). Immunohistochemical staining of the islets showed no significant differences between the two groups in terms of the area or intensity of α- and β-cell staining for insulin and glucagon.

**Conclusions:**

To our knowledge, this is the first report of the effect of intermittent hypoxia on UCP2 expression. Our findings suggest that UCP2 regulates insulin production in OSA. Further study of the role that UCP2 plays in the glycemic control of OSA patients is warranted.

## Introduction

Obstructive sleep apnea (OSA) is characterized by recurrent collapse of the pharynx during sleep. Each apneic event results in oxygen desaturation and, occasionally, arousal, causing intermittent hypoxia, oxidative stress, and sleep fragmentation. ^(^ 1 ^)^ Up to 32% of the adult population is affected by OSA.^(^ 2 ^)^

Various cardiovascular and metabolic outcomes have been attributed to OSA.^(^ 3 ^)^ Metabolic syndrome shares several features with OSA, including obesity, hyperlipidemia, hypertension, and insulin resistance, which might be implicated in an increased risk of cardiovascular disease.^(^ 4 ^-^ 6 ^)^ In patients with OSA, metabolic syndrome can be improved by treatment with continuous positive airway pressure,^(^ 7 ^-^ 9 ^)^ indicating a causal relationship between the two conditions.

Experimental models that subject animals to intermittent hypoxia are used in order to study the consequences of OSA. Insulin resistance and metabolic changes have been reported after exposure to such models.^(^ 10 ^,^ 11 ^)^ The mechanisms underlying the changes in glucose metabolism induced by intermittent hypoxia have yet to be fully explored.

Uncoupling protein-2 (UCP2) is a negative regulator of pancreatic β-cell insulin secretion. ^(^ 12 ^-^ 14 ^)^ The UCPs, located in the mitochondrial inner membrane, translocate protons from the intermembrane space to the mitochondrial matrix. The protons that leak through a UCP can no longer be used to drive the rotation of ATP synthase, such proton leakage thus decreasing the generation of ATP. By abating ATP synthesis, UCP2 regulates glucose-stimulated insulin secretion.^(^ 15 ^)^ Free radicals generated during hypoxia-reoxygenation in sleep apnea may play a regulatory role in the pancreatic β-cells.^(^ 16 ^)^ This is the rationale for investigating a possible apnea-UCP2 relationship.

Because of the epidemic proportions that diabetes and OSA are assuming, there is interest in understanding the interplay between these two conditions. In an extensive search of the literature, we found no reference to the molecular regulation of insulin secretion in OSA. We hypothesized that intermittent hypoxia would change the expression of UCP2. Therefore, we investigated the effect of intermittent hypoxia on pancreatic UCP2 expression in C57BL mice.

## Methods

### Animals and intermittent hypoxia

We evaluated 36 male C57BL mice, in two groups: exposed to intermittent hypoxia (hypoxia group, n = 18); and subjected to a control (sham) procedure (normoxia group, n = 18). All of the mice were 8-9 weeks of age at the start of the study. All procedures were conducted in accordance with the Guide for the Care and Use of Laboratory Animals,^(^ 17 ^)^ and the study protocol was approved by the Research Ethics Committee of the Porto Alegre *Hospital de Clínicas*, in the city of Porto Alegre, Brazil (Protocol no. 09-300).

Groups of 6 mice were housed under temperature-controlled conditions (22.5-24.5ºC), on a 12/12-h light/dark cycle (lights on at 7:00 a.m.), and provided with standard mouse chow and water *ad libitum*. Using a precision balance with a resolution of 0.01 g (AS 5500C; Marte Científica, São Paulo, Brazil), we weighed the mice at baseline and every 3 days thereafter, until the end of the experiment. The food pellets in each cage were weighed daily, in order to quantify food intake.

Over a period of 35 days, for 8 h a day during the lights-on period (from 9:00 a.m. to 5:00 p.m.), the mice were placed in the intermittent hypoxia system. In the intermittent hypoxia condition, a gas mixture consisting of 92% nitrogen and 8% CO_2_ was released into the hypoxia chamber for 30 s, progressively reducing the fraction of inspired oxygen (FiO_2_) from 21% to a nadir of 8 ± 1%. Next, a fan moved room air into the chamber for 30 s, restoring the FiO_2_ to 21% within 5 s. Therefore, each hypoxia/normoxia cycle lasted for 60 s (60 cycles/h), for a total of 480 hypoxia cycles over the 8-h period. In the control (normoxia) condition (sham procedure), the mice were housed in an adjacent cage and were exposed to the same fan-off/fan-on cycles, although no gas mixture was released into the chamber at any point.

At the end of the experimental period, the animals were fasted for 6-10 h, after which they were anesthetized by intraperitoneal injection of ketamine (100 mg/kg) and xylazine (10 mg/kg). After anesthesia was confirmed, blood samples were collected from the retro-orbital sinus. The mice were exsanguinated during the procedure. Blood samples were centrifuged at 10,000 rpm for 10 min at 4ºC to obtain serum samples. The pancreases of 12 mice per group were dissected, extracted, soaked for 24 h in five volumes of tissue storage reagent (RNAlater^(r)^; Applied Biosystems/Ambion, Austin, TX, USA), and stored at 4ºC overnight, after which they were frozen at −80ºC until analysis. The pancreases of 6 mice per group were fixed in 10% formalin for 24 h and embedded in paraffin for future processing.

### Real-time PCR for UCP2, islet immunohistochemistry, and biochemical assays

The pancreases were unfrozen, dissected to isolate the islets, and homogenized in optimized phenol/guanidine-based lysis solution. Total RNA was isolated using TRI reagent solution (Applied Biosystems/Ambion). We quantified RNA and tested its quality by photometric measurement on a spectrophotometer (ND-1000; NanoDrop Technologies, Wilmington, DE, USA). Using a High Capacity cDNA Reverse Transcription Kit (Applied Biosystems, Foster City, CA, USA), we synthesized cDNA from 2 µg of highly purified RNA (A260/A280>1.95). The cDNA was stored at −20ºC and diluted in DNase-free water (1:100) before relative quantification by real-time PCR. Gene expression analysis was performed using TaqMan Gene Expression Assays for the UCP2 gene (Mm00627598_m1; Applied Biosystems), using the following primer sequences: forward, 5′-ACAAGACCATTGCACGAGAG-3′ and reverse, 5′-ATGAGGTTGGCTTTCAGGAG-3′. The results were normalized to glyceraldehyde-3-phosphate dehydrogenase (GAPDH) expression. Thermal cycling was performed in a real-time PCR system (StepOne(tm); Applied Biosystems). 

Gene expression was quantified using the 2-ΔΔCt (threshold cycle) method.^(^ 18 ^)^ Each sample was analyzed in duplicate, and the ΔCt value was obtained by subtracting the GAPDH Ct value from the Ct value of the UCP2 gene. To calculate the difference between groups, the mean ΔCt value obtained for the control group was used to calculate the ΔΔCt of the gene (2-ΔΔCt).

The paraffin-embedded pancreases were sectioned into 5-µm slices. Sections were mounted on glass slides, coated with 3-aminopropyltriethoxysilane, deparaffinized with xylene (two changes, 5 min each), and rehydrated in a graded ethanol series (four successive washes), after which they were immersed in distilled water and PBS. For antigen retrieval, the slides were immersed in 10 mM sodium citrate buffer, pH 6.0, heated in a microwave oven for 21 min and cooled to room temperature. To inhibit endogenous peroxidase activity, the slides were twice immersed in 5% hydrogen peroxide for 15 min. Nonspecific antibody-binding sites were blocked with 5% nonfat dry milk diluted in PBS for 20 min. Immunohistochemistry staining for β- and α-cells was performed using polyclonal guinea pig anti-insulin (A0564, 1:200 dilution; Dako, Carpinteria, CA, USA) and rabbit polyclonal antibody to glucagon (GA 1181, 1:4,000; Enzo Life Sciences, Farmingdale, NY, USA), respectively. After three washes, in running water, distilled water, and PBS, respectively, the slides were incubated for 1 h in a humidity chamber at room temperature. They were then washed once with distilled water and three times with PBS, after which they were incubated with a secondary antibody (Picture-MAX Polymer Kit; Invitrogen, Camarillo, CA, USA) for 30 min in the humidity chamber. We developed the slides using 3,3'-diaminobenzidine as chromogen, according to manufacturer instructions. The slides were then counterstained with Harris's hematoxylin for 20 s, washed in running water, and rinsed with ammonium hydroxide to obtain a light blue color. They were subsequently washed once with water, twice with ethanol, and three times with xylene. All sections were analyzed under light microscopy (magnification, ×400), digitized, and assessed with a computer image analysis system (Image-Pro Plus, version 6.0; Media Cybernetics, Bethesda, MD, USA). The area of β-cells in the islets was determined by the insulin-positive cell area, which was calculated as the ratio of intra-islet insulin-positive area to total islet area. The relative area of glucagon, which represented the amount of α-cells in each islet, was evaluated by the same method. The insulin positive- and glucagon positive-cell indices for each group were calculated as the average of at least 24 sections. Nuclei were not taken into account for quantitative analysis of islet cell composition.

Fasting serum glucose, total cholesterol, HDL cholesterol, and triglycerides were measured by enzymatic colorimetric methods using commercially available kits (Labtest Diagnostics, Belo Horizonte, Brazil). We estimated LDL cholesterol levels by the Friedewald formula^(^ 19 ^)^: 

*LDL cholesterol* = (*total cholesterol* − *HDL cholesterol* − *triglycerides) *∕ 5

Fasting serum insulin was determined using a mouse insulin ELISA kit (ALPCO Diagnostics, Salem, NH, USA). Fasting serum glucagon was quantified with a glucagon enzyme immunoassay (EIA) kit (Phoenix Pharmaceuticals, Burlingame, CA). To evaluate the degree of insulin resistance, we calculated the homeostasis model assessment of insulin resistance (HOMA-IR) value, using the following formula^(^ 20 ^)^: 

*HOMA-IR* = *fasting serum insulin* (µU/mL) × *fasting blood glucose* (mmol/L) ∕ 22.5

In addition, we calculated the HOMA of pancreatic β-cell function (HOMA-β), as follows:

*HOMA-*β = [*fasting serum insulin level* (µU/mL) × 20] ∕ [*fasting blood glucose level* (mmol/L) − 3.5]

### Statistical analysis

Data were tabulated and expressed as medians and interquartile ranges and in box plots showing 10th, 25th, 50th, 75th, and 90th percentiles, as well as outliers. The Mann-Whitney U test was used for between-group comparison of median UCP2 mRNA, insulin/glucagon-positive cell area, and biochemical assay values. Body weight in each group was adjusted for food intake using the generalized estimating equations (GEE) method, followed by pairwise comparisons with sequential Bonferroni correction. All statistical analyses were performed with the Statistical Package for the Social Sciences, version 17.0 for Windows (SPSS Inc., Chicago, IL, USA). Statistical significance was assumed if the probability of alpha error was < 0.05.

## Results

**Figure 1 -:**
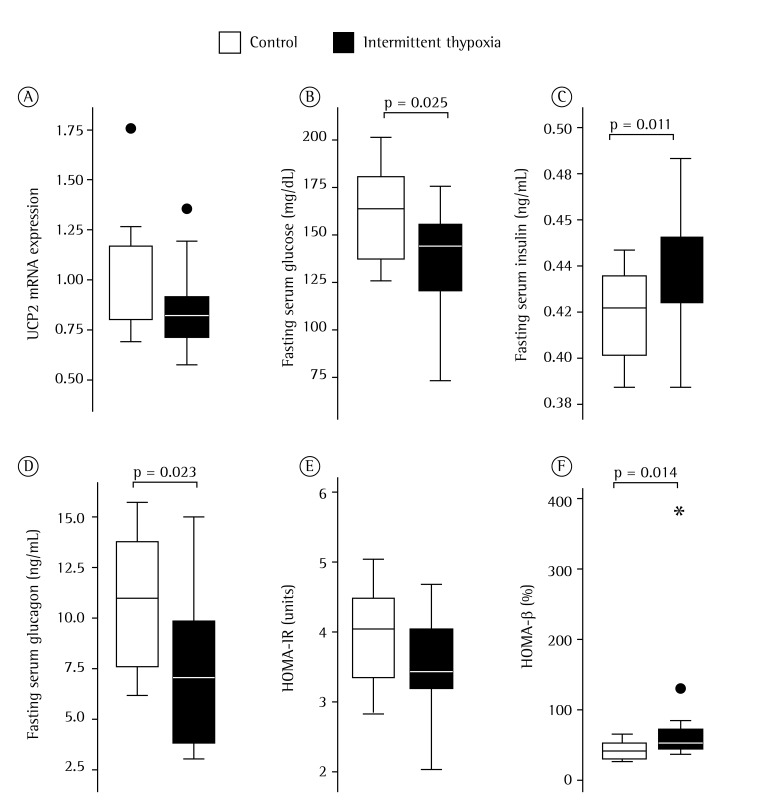
Box plots of glucose metabolism variables showing 10th, 25th, 50th, 75th, and 90th percentiles; outliers (filled circles); and extreme outliers (asterisk). UCP2: uncoupling protein-2; HOMA-IR: homeostasis model assessment of insulin resistance; and HOMA-β; homeostasis model assessment of pancreatic β-cell function. Values of p were calculated by the Mann-Whitney U test.

Data showing the expression of UCP2 in both groups are displayed in Figure 1A. The relative expression of UCP2 was 16% lower in the hypoxia group than in the normoxia group, (0.82 [0.71-0.94] vs. 0.98 [0.78-1.19]), although the difference between the two groups was not significant (p = 0.11).

Fasting serum glucose levels (Figure 1B) were 12% lower in the hypoxia group than in the normoxia group (144 [115-156] vs. 163 [133-184] mg/dL; p = 0.025). Fasting serum insulin levels (Figure 1C) were 5% higher in the hypoxia group as compared with the normoxia group (0.44 [0.42-0.46] vs. 0.42 [0.40-0.44] ng/mL; p = 0.01). Fasting serum glucagon levels (Figure 1D) were 37% lower in the hypoxia group than in the normoxia group (6.84 [3.64-9.90] vs. 10.87 [7.43-14.21] ng/mL; p = 0.023).

After exposure to intermittent hypoxia, the mean HOMA-IR value (Figure 1E) was 15% lower in the hypoxia group than in the normoxia group, although the difference was not significant (3.45 [3.00-4.17] vs. 4.06 [3.35-4.55]; p = 0.095). The mean HOMA-β value (Figure 1F) was 21% higher in the hypoxia group than in the normoxia group (46.6 [42-74] vs. 36.6 [29-52]; p = 0.014).

**Figure 2 -:**
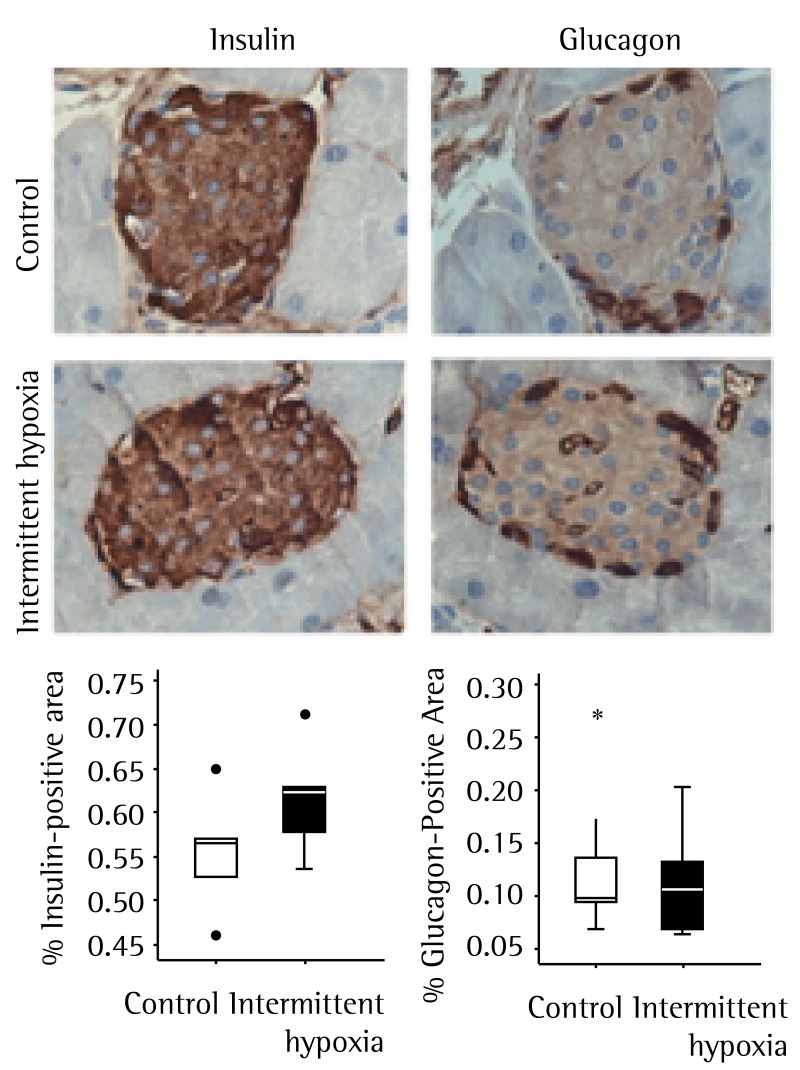
Immunohistochemical staining for insulin and glucagon (magnification, ×400) with box plots of the relative area of the islet occupied by β-cells and α-cells. Box plots show 10th, 25th, 50th, 75th, and 90th percentiles; outliers (filled circles); and extreme outliers (asterisk).

As shown in Figure 2, the relative area of insulin-positive cells in the hypoxia group was non-significantly higher than in the normoxia group: 0.62 [0.56-0.67] vs. 0.56 [0.51-0.59] respectively (p = 0.14). The area of glucagon-positive cells was 0.10 [0.06-0.15] in the hypoxia group and 0.09 [0.08-0.17] in the normoxia group (p = 0.67).

Lipid profile parameters were compared between groups. Mice exposed to intermittent hypoxia did not differ significantly from the control mice in terms of fasting serum triglycerides or the levels of HDL, LDL, and total cholesterol.

Body weights remained relatively stable over the 35-day study period: 23.7 ± 0.3 g at day 1 versus 25.1 ± 0.3 g at day 35 in the normoxia group; and 24.6 ± 0.5 g at day 1 versus 24.8 ± 0.3 g at day 35 in the hypoxia group. Body weight entered as a dependent variable in the GEE model showed no effect of group allocation or of food intake. Time had a significant effect, and there was a significant interaction between time and group (p < 0.001 for both).

## Discussion

To our knowledge, this was the first study to analyze the effect of an intermittent hypoxia-based model of OSA on the expression of UCP2 mRNA in the pancreas. In this model, exposure to 480 cycles per day of intermittent hypoxia with an 8% FiO_2_ nadir for 35 days resulted in levels of UCP2 mRNA expression lower than those observed for the control condition, although the difference was not statistically significant. These preliminary results supplement the current knowledge of the mechanisms underlying the metabolic consequences of OSA, generating various hypotheses. The lack of a significant reduction in UCP2 expression in the hypoxia group might indicate that intermittent hypoxia has no effect on the expression of UCP2 or that it downregulates that expression to a degree below the threshold of the statistical power conveyed by the small number of animals evaluated.

The results of the present study add to the debate on the metabolic changes induced by OSA. Our data show that mice exposed to intermittent hypoxia had lower fasting serum glucose and glucagon levels than did the control mice. Previous studies have demonstrated that mice exposed to intermittent hypoxia show glucose levels that are either elevated^(^ 10 ^,^ 21 ^)^ or lowered. ^(^ 11 ^)^ Our findings agree with those of the latter study, which involved lean and obese C57BL/6J mice exposed to chronic intermittent hypoxia for 5 days.^(^ 11 ^)^ In the lean mice, glucose levels and insulin resistance decreased after hypoxia. In the obese (leptin-deficient) mice, glucose levels also decreased, although insulin resistance increased.^(^ 11 ^)^ In the present study, only lean mice were employed, and our results might therefore represent a true response of lean mice exposed to intermittent hypoxia as evidenced by the increased insulin levels and decreased levels of glucagon.

Our finding that fasting serum insulin levels and β-cell function were higher in the hypoxia group than in the normoxia group is consistent with the lower glucose and glucagon levels previously reported as a response to hypoxia. In previous studies, obese mice exposed to intermittent hypoxia showed higher insulin levels than did control mice,^(^ 11 ^,^ 21 ^,^ 22 ^)^ although the FiO_2_ and exposure period differed from those employed in the present study. Another study showed that insulin levels were lower in lean mice exposed to intermittent hypoxia than in control mice.^(^ 23 ^)^

In the present study, insulin levels were higher in mice exposed to intermittent hypoxia than in the control mice. The significantly lower levels of glucagon found in the hypoxia group might indicate that compensatory mechanisms are activated to maintain glucose homeostasis. A review of the literature found no comparable studies reporting the response of glucagon to intermittent hypoxia.

No differences in insulin resistance were detected by HOMA-IR in the mice subjected to intermittent hypoxia, their glucose levels being lower than were those of controls. The HOMA-β value was higher in the former group, suggesting that some mechanism-perhaps a lowering of UCP2 levels-was activated in order to increase insulin production. The HOMA-IR and HOMA-β have not been validated for use in rodents, the formula for the latter representing a measure of β-cell function in humans.^(^ 24 ^)^ However, the difference in the insulin to glucose ratio suggests that the mice might become insulin resistant, as previously reported.^(^ 11 ^)^

To date, there have been few reports in the literature addressing pancreatic β-cell function and sleep. In the experiment reported here, the HOMA-β values were significantly elevated in the animals exposed to intermittent hypoxia for 35 days, being 10% higher in the hypoxia group than in the normoxia group. The increase in the HOMA-β value implies that OSA causes an excessive increase in the functional demand on pancreatic β-cells, which leads to worsening of insulin secretion as the disease progresses.

Our findings should be interpreted with caution. The data related to glucose levels might be unreliable, because even the normoxia group mice had high glucose levels, despite fasting. One possible explanation for this, other than an error in measurement, is that, although the food pellets were removed from the cages, a significant ration could have remained inside the cage. However, the error might have been systematic, the lower glucose level in the hypoxia group being an effect of the higher insulin level. Although the result is negative, further exploration of the effect of intermittent hypoxia on UCP2 could be useful. Models employing UCP2 knockout mice could clarify the actual reaction of this protein to intermittent hypoxia. An oral glucose challenge would confirm insulin resistance, and a curve would be more informative than are single measurements. Although difficult in mice, this has been done previously.^(^ 6 ^)^ The low triglycerides suggest that the hypoxia group mice were not eating as much as were the normoxia group mice. This could be a potential confounder of the findings.

Hyperlipidemia is common in persons with sleep apnea. Experiments evaluating the effect of intermittent hypoxia on lipid profile have reported discordant findings due to inconsistency in methods among studies. Examples of parameters that vary from study to study and could account for discrepant findings are differences in the duration of exposure (in days and in hours per day), in the duration of each hypoxia cycle (in seconds), in the FiO_2_ nadirs, and in mouse strains. The magnitude of the metabolic disorder induced by intermittent hypoxia depends on the nadir of FiO_2_. Experiments using 10% FiO_2_ as the nadir of the hypoxia cycle did not induce metabolic changes after 28 days. Conversely, using a 5% FiO_2_ nadir led to changes in all lipid profile parameters.^(^ 25 ^)^ The moderate severity of intermittent hypoxia-both in terms of FiO_2_ nadir and of hours of exposure per day-might be one reason for the absence of changes in lipid parameters in the present study. Similarly, the 8% FiO_2_ nadir and 8 h of hours of exposure per day employed here might not have induced hypoxia severe enough to affect UCP2 expression.

In conclusion, our investigation of the effect of intermittent hypoxia on the expression of UCP2 mRNA in C57BL mice revealed that, after 35 days of exposure to intermittent hypoxia, there was no significant change in the expression of this regulatory protein. At the level of severity employed here, intermittent hypoxia causes changes in pancreatic function that might be unrelated to potential changes in the expression of UCP2 mRNA.
